# Evidence for Specific Associations Between Depressive Symptoms, Psychotic Experiences, and Suicidal Ideation in Chilean Adolescents From the General Population

**DOI:** 10.3389/fpsyt.2020.552343

**Published:** 2021-01-28

**Authors:** Daniel Núñez, Pía Monjes, Susana Campos, Johanna T. W. Wigman

**Affiliations:** ^1^Faculty of Psychology, Universidad de Talca, Talca, Chile; ^2^Millennium Nucleus to Improve the Mental Health of Adolescents and Youths, Imhay, Santiago, Chile; ^3^Programa de Investigación Asociativa, Faculty of Psychology, Centro de Investigación en Ciencias Cognitivas, Universidad de Talca, Talca, Chile; ^4^Department of Psychiatry, University Medical Center Groningen, University of Groningen, Groningen, Netherlands

**Keywords:** suicide, adolescents, network and mediation analysis, depressive symptoms, psychotic experiences

## Abstract

Associations between psychotic experiences and suicidal ideation are not yet fully understood, and the potential role of depressive symptoms in this relationship remains unclear. The current study examined relationships between depressive symptoms (DS), psychotic experiences (PE) and suicidal ideation (SI) using two complementary approaches on cross-sectional data from a community sample of adolescents aged 13–19 years (*N* = 1,591). First, we investigated the association between the three domains using mediation analysis, showing that depressive symptoms partly mediate the relationship between psychotic experiences and suicidal ideation. Second, we looked at associations between the three domains at item level using network analysis. Specific associations between symptoms of the three domains were found, indicating depressive symptoms of sadness, avolition, pessimism, and self-criticalness/worthlessness as the most central symptoms in the network. Suicidal ideation was associated with the depressive symptoms pessimism and worthlessness, to social anxiety, and to perceptual anomalies. Our results show that the mediating effect of depressive symptoms between psychotic experiences and suicidal ideation may be due to associations between specific aspects of SI, depressive symptoms and psychotic experiences. These findings can contribute to the planning of health services and programs aimed at the timely detection of psychopathology and suicidal risk in young people.

## Introduction

Youth mental health is a global challenge, with onset of mental illness peaking in adolescence (1). Adolescents are at increased risk for both psychotic experiences (PE) (2) and suicidal ideation (SI) (3) which are prevalent in the general (4) and clinical populations (5). Both are associated with psychological distress and higher prevalence of mental disorders (6) and are regarded as early markers for subsequent psychiatric vulnerability in adolescents (7–9). Especially the combination of the two seems problematic in this young population. Suicidal risk has been associated with PE (10) in general populations (11, 12) and clinical samples (13, 14), particularly when PE are persistent (15, 16). However, the underlying mechanisms explaining the links between PE and SI remain uncertain (17, 18). Some researchers posit that the association between PE and SI is independent of third variables (19, 20), and other argue that the relationship is influenced by third variables such as shared risk factors (21) or mental distress (22).

One factor that has been suggested as a potential mechanism between PE and SI is depressive symptoms (DS) (23). Depressive symptoms are highly prevalent in adolescents (24) and have been proposed as a potential underlying mechanism between PE and SI (25). Even at subthreshold levels, DS are associated with poor social well-being (26), social and educational impairments (27) and increased risk for psychopathology and suicide (28). Literature also supports strong associations between DS and PE (29) suggesting that they are interwoven co-occurring phenomena at both clinical and subclinical levels (30, 31).

Taken together, there is ample evidence suggesting that the three domains of PE, DS, and SI are closely intertwined. However, it remains unclear how exactly they are associated. According to Granö et al. (32), visual distortions could explain SI in adolescents; even when depressive symptoms (and other psychotic symptoms) were controlled for. Fujita et al. (14) reported that auditory verbal hallucinations, but not depressive symptoms, increased the risk for suicidal attempts in adolescents with suicidal ideation. Gill et al. (5) found that the associations between negative symptoms and SI persisted when controlled for depressive symptoms in a high-risk sample of adolescents and young-adults, and Nishida et al. (33) observed that the association between PE and suicidal feelings and behaviors remains significant when controlled for anxiety and DS and use of substances in a general sample of adolescents. Similar results were found in help-seeking adolescents (3). By contrast, Sullivan et al. (34), observed that PE and DS were independently associated with suicidal behavior (SB), and that the association with DS was substantially stronger. Additionally, they reported that PE alone were not a strong predictor of later SB when compared with the predictive power of DS. According to Hielscher et al. (35), the associations between delusional experiences and non-accidental self-injuries were non-significant when depression was entered into the model, which supports prior studies revealing that depressive symptoms mediate the association (12).

Disentangling the associations among PEs, SI, and DS could be helpful to better understand the trajectories and clinical outcomes of these overlapping phenomena. Timely evaluations and interventions to reduce their severity might decrease their impact. Intensity of depression is associated with poorer outcomes of PE in both subjects at risk for psychosis (36) and general population (37), and lower depression levels are associated with reductions in PEs in high risk subjects (38). We currently know that lower DS has better outcomes for PE (39) and that less stressful PE has lower risk for SI (40). However, if we want to know which strategy would be most helpful, we need to disentangle the routes of association between these three phenomena in more detail.

In sum, PE, DS as well as SI are (i) common in adolescents, (ii) by themselves indicative of mental health problems, and (iii) risk factors for further psychopathological development. However, the role of DS within the SI-PE associations remains controversial (35). Therefore, we examined relationships among them using two complementary approaches. First, to explore the associations among these variables, we conducted regression-based mediation analyses (41), hypothesizing that DS would mediate the link between PEs and SI. Second, we hypothesized that a potential mediating role might consist of specific associations among particular subtypes of PE, SI, and DS (29) and investigated this using network analysis, which allows for the exploration of interactions among specific symptoms (42). We hypothesized that DS would be a mediating role in the association between PE and SI. This role of DS seems to be adequate considering that SI is one of the more severe symptoms of depression (43), being the latter a predictor of SI and suicide attempts (44), but not vice versa. We expected to find positive relationships among SI, PE, and DS. In line with previous findings (45, 46) we hypothesized that DS would take up a central role in the overall symptoms network, and that some specific affective symptoms (i.e., low energy, hopelessness and self-depreciating feelings) would show higher centrality values (i.e., play a more central role in the symptom network).

## Method

### Participants

We conducted a cross-sectional study with adolescents recruited between April and August 2015 in 6 Chilean public secondary public schools in the urban area of the city of Talca. One thousand seven hundred and seventy three students and their parents voluntarily agreed to participate in the study and provided written informed consent. We excluded 182 subjects who did not complete all measures. Little's test (45) showed that these excluded individuals did not differ from included participants on demographic (gender and age) or clinical severity (total score on depressive, anxiety and stress symptoms (DASS-21). We performed the analyses with a final sample of 1591 adolescents (mean age = 16.01, SD = 1.45, women = 53.4%).

### Measures

#### Psychotic Experiences (PE)

As previously reported (47), we used a deductive method of item generation (48) where we combined two pre-existing scales that were used in prior studies with adolescents: the Community Assessment of Psychic Experiences—Positive scale [CAPE-P15 (49)], and the Brief Self-report Questionnaire for Screening Putative Pre-psychotic States [BQSPS (50)]. We assessed the following domains: bizarre experiences (BE, 6 items), perceptual anomalies (PA, 3 items), social anxiety (SA, 3 items), and negative symptoms (NS, 3 items). Both reliability and internal consistency were adequate when assessed through the coefficients of Cronbach's alpha and Macdonald's Omega [i.e., ω > 0.65 (51); Table 1].

**Table 1:** Internal consistency values for each subscale.

**Dimension**	**Cronbach's α**	**McDonald's ω**
Bizarre experiences	0.78	0.78
Perceptual anomalies	0.79	0.81
Social anxiety	0.68	0.69
Negative symptoms	0.65	0.69
Suicidal ideation lifetime	0.85	0.87
Depression	0.89	0.90

#### Depressive Symptoms (DS)

We assessed DS with the Depression and Anxiety Scale (DASS-21) (51), a 21-item self-report questionnaire with three subscales assessing depressed mood, anxiety and stress. In the present study, we only used the subscale for depressive symptoms (DS). The reliability of the instrument is good for this sample (Cronbach's alpha = 0.89; McDonal's ω = 0.90) (Table 1).

#### Suicidal Ideation (SI)

We assessed SI using 6 items of the Columbia Suicide Severity Rating Scale (C-SSRS) (52), adapted for being used as a self-report questionnaire (53). Severity of SI was rated on a 6-point ordinal scale in which 1 = wish to be dead, 2 = non-specific active suicidal thoughts, 3 = thoughts about how to commit suicide, 4 = suicidal thoughts and intentions, 5 = suicidal thought with detailed plan, and 6 = intentions to conduct plan. Frequency of SI was addressed by asking participants when these thoughts happened: ever in life (SI_L_) and/or during last month (SI_M_). We only reported the former (SI_L_) because there were few reports of suicidal ideation during the last month. The internal consistency of SI_L_ is good for the current population (Cronbach's alpha = 0.85; McDonal's ω = 0.87).

### Procedure

The translating process of the questionnaires has been previously described (47). We conducted the study in public schools who agreed to participate after meetings with directive committees. Researchers participated in different parents' meetings to present the project. After its approval and once written informed consents were obtained from both adolescents and their caregivers, the participants completed the questionnaires, administered in classroom settings by trained psychologists. Ethical approval was obtained from the Bioethics Committee of the University of Talca.

### Data Analysis

Prior to the main analysis, we first examined the associations among the variables, using Spearman's correlation coefficient. To answer the first research question of the indirect effect of depressive symptoms on the relationship between PE and SI, we conducted a mediation analysis through the modeling macro PROCESS v.2.13 (54). This statistical package uses least squares regression-based path analytic framework for mediation analysis that follows the Baron and Kenny procedure (41). This analysis was carried out using the bootstrapping resampling method through 5,000 bootstrap resamples (55). An advantage of the bootstrapping approach for this analysis is that it overcomes the sample size requirements used by the Sobel test to assess mediation (56, 57). Bootstrapping resampling estimates the indirect effect and its 95% confidence intervals. When the bias-corrected confidence intervals (BC CIs) do not contain 0, it is assumed that there is a mediating effect among the proposed variables. In this model, the predictor variable was PE, the outcome variable was SI, and the mediator variable was DS. No other variables were controlled for. Additionally, to investigate whether the mediating role of DS was not explained by more general distress, we performed two single mediation models, where the mediators between PE and SI were anxiety (AS) in the first model and stress symptoms (S) in the second model. Finally, we computed a multiple mediation model testing DS, AS and S as mediators between PE and SI.

Next, to examine the second research question on simultaneous relationships among single items, we used network analysis. We estimated an Ising network model for binary data, previously used to analyze psychopathology (58). The model assumes that the activation of a node depends on the activation of its neighboring nodes. The binary network was fitted using the R-package IsingFit 0.3.1. For model estimation, as previously done (46), we recoded the responses of questionnaires as follows: For PE: 0 = “not present” (scores 1–2), 1 = “present” (scores 3–5). For SI: 0 = “not present” (scores 1–2), 1 = “present” (scores 3–6). For DS: 0 = “not present” (1, 2), 1 = “present” (3–5).

After the estimation of the Ising model, we examined the resulting network using the qgraph package, version 1.6.3. Given recent concerns on the suitability of using centrality indices when applying network analyses to psychopathology, and following the suggestion of Bringmann et al. (59), we focused only on the strength centrality index as a measure of relative importance in the whole network. Strength is defined as the sum of the weights of the links of a node with other nodes and represents how well-connected a node is to the rest of the network (44). We eliminated spurious associations between nodes and excluded small associations from the graphs using the graphical LASSO (Least Absolute Shrinkage and Selection Operator) implemented in the R package q graph (60). Next, we analyzed the network stability by the correlation stability coefficient (CS-coefficient), which quantifies the maximum amount of cases that can be dropped to retain with 95% certainty, a correlation with the original centrality of higher than (by default) 0.7. Values should be at least 0.25 for the centrality to be stable, and preferably above 0.5 (60). Finally, we tested the accuracy of the edges in the network by bootstrapping the 95% confidence intervals of the edge weights to test if the edges do significantly differ from one-another.

## Results

### Descriptive Statistics

Tables 2–5 show raw and dichotomized scores of, respectively, PE, DS, and SI. Of the PE, NS had the highest prevalence (69.2%), followed by SA (63.2%), BE (52.5%), and PA (15.3%). The prevalence of DS was 50.4%. The prevalence of SI was 19%.

**Table 2:** Prevalence (%) of psychotic experiences, depressive symptoms, and suicide ideation in the study sample (at least one time, lifetime).

	**Psychotic experiences**				**DS**	**SI**
	**BE**	**PA**	**SA**	**NS**		
Males	340 (45.8)	99(13.3)	441 (59.4)	503 (67.7)	309 (41.6)	80 (10.8)
Females	497 (58.5)	144 (16.9)	565 (66.5)	599 (70.5)	494 (58.1)	223 (26.2)
Total	837 (52.5)	243 (15.3)	1006 (63.2)	1102 (69.2)	803 (50.4)	303 (19)

*PE, Psychotic experiences (Total); BE, Bizarre experiences; PA, Perceptual anomalies; SA, Social anxiety; NS, Negative symptoms; DS, Depressive symptoms; SI, Suicidal ideation*.

**Table 3:** Descriptive scores of psychotic experiences (raw and dichotomized scores).

**Item**	**Domain**	**Mean scores (SD)**		**Median**	**%**	
		**Original responses**	**After recoding**		**Yes**	**No**
Have you ever felt that you are being persecuted in anyway?	BE1	1.78 (0.95)	0.20 (0.399)	2	19.90	80.10
Have you ever felt as if there is a conspiracy against you?	BE2	1.63 (0.91)	0.15 (0.355)	1	14.87	85.13
Have you ever felt that people look at you oddly because of your appearance?	BE3	2.01 (1.08)	0.26 (0.439)	2	26.00	74.00
Have you ever felt as if the thoughts in your head are being taken away from you?	BE4	1.68 (0.93)	0.18 (0.381)	1	17.65	82.35
Have you ever felt as if the thoughts in your head are not your own?	BE5	1.54 (0.88)	0.13 (0.334)	1	12.70	87.30
Have your thoughts ever been so vivid that you were worried other people would hear them?	BE6	1.72 (1.05)	0.19 (0.396)	1	19.39	80.61
Have you ever heard voices when you are alone?	PA1	1.46 (0.86)	0.11 (0.312)	1	11.49	88.51
Have you ever seen objects, people or animals that other people can't see?	PA2	1.24 (0.68)	0.06 (0.243)	1	6.30	93.70
I feel I cannot get close to people	SA1	1.80 (1.03)	0.21 (0.404)	1	20.80	79.20
I am mostly quiet when with others	SA2	2.12 (1.09)	0.31 (0.462)	2	30.72	69.28
I feel nervous when giving a speech in front of a large group of people	SA3	2.79 (1.33)	0.53 (0.499)	3	52.80	47.20
I cannot focus on a task	NS1	2.70 (1.17)	0.51 (0.500)	3	51.70	48.30
I feel mentally insufficient and easily fatigued while thinking or reading	NS2	1.78 (0.98)	0.17 (0.382)	2	17.17	82.83
I need to take frequent breaks while working (studying)	NS3	2.69 (1.11)	0.51 (0.500)	3	50.90	49.10

*BE, Bizarre experiences; PA, Perceptual anomalies; SA, Social anxiety; NS, Negative symptoms*.

**Table 4:** Descriptive scores of depressive symptoms (raw and dichotomized scores).

**Item**	**Mean scores (SD)**		**Median**	**%**	
	**Original scores**	**After recoding**		**Yes**	**No**
I couldn't seem to experience any positive feeling at all	1.80 (1.06)	0.21 (0.41)	1	21.47	78.53
It was difficult for me to motivate myself to do things	2.12 (1.15)	0.30 (0.46)	2	30.50	69.50
I felt that I had nothing to look forward to	1.45 (0.98)	0.12 (0.33)	1	12.20	87.80
I felt down-hearted and blue	2.14 (1.26)	0.32 (0.47)	2	32.59	67.41
I was unable to become enthusiastic about anything	1.62 (0.94)	0.14 (0.35)	1	14.74	85.26
I felt I wasn't worth much as a person	1.69 (1.14)	0.19 (0.39)	1	19.64	80.36
I felt that life was meaningless	1.58 (1.10)	0.16 (0.37)	1	16.30	83.70

**Table 5:** Descriptive scores of suicidal ideation lifetime (dichotomized scores).

**Item**	**Mean scores (SD)**	**Lifetime**	
		**%**	
		**Yes**	**No**
Have you wished you were dead or wished you could go to sleep and not wake up?	0.47 (0.499)	47.17	52.83
Have you actually had any thoughts about killing yourself?	0.15 (0.356)	14.93	85.07
Have you thought about how you might do this?	0.21 (0.406)	20.80	79.20
Have you had any intention of acting on these thoughts of killing yourself,	0.18 (0.386)	18.20	81.80
Have you started to work out or worked out the details of how to kill yourself?	0.06 (0.237)	6.00	94.00
Do you intend to carry out this plan?	0.05 (0.216)	4.90	95.10

### Correlation Analysis

Spearman's correlations are displayed in Supplementary Table 1. DS were significantly correlated with SI (*r* = 0.49, *p* < 0.001), with PE (*r* = 0.63, *p* < 0.001) and, specifically, PE factors BE (*r* = 0.52, *p* < 0.001), SA (*r* = 0.44, *p* < 0.001), NS (*r* = 0.43, *p* < 0.001). PA (*r* = 0.35, *p* < 0.001). Likewise, PE and SI were significantly linked (*r* = 0.43, *p* < 0.001).

### Mediation Analysis

**Figure 1:**
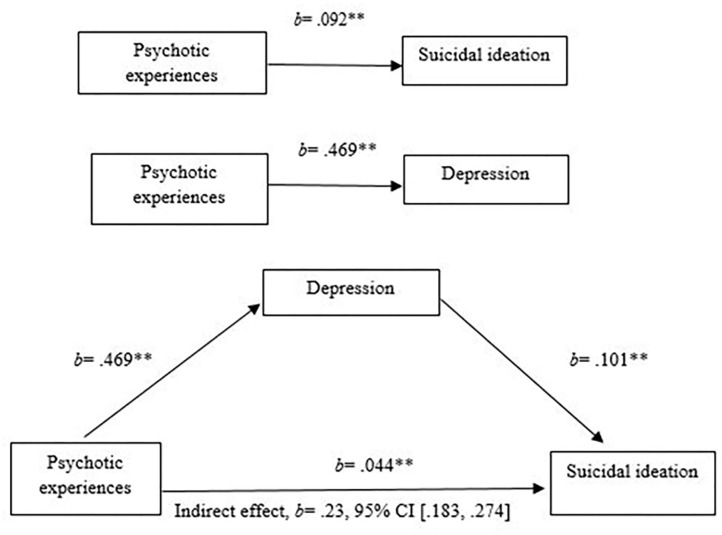
Mediation analysis of the relationship of psychotic experiences and suicidal ideation mediated by depressive symptoms.

We observed positive and significant associations amongst PE and DS, DS and SI, and PE and SI, and a significant indirect effect for DS. This means that DS effectively mediates the association between PE and SI (b = 0.227, 95% BCa CI [0.183, 0.274]) (Table 6 and Figure 1). When DS were added in the model, the association between PE and SI remained significant, suggesting a partial mediation. PE accounted for medium to high amounts of variance in suicidality, with *R*^2^ = 0.44 and in depressive symptoms, with *R*^2^ = 0.63.

**Table 6:** Mediation analysis of psychotic experiences, depressive symptoms, and suicidal ideation.

**Mediation steps**	**Outcome**	**Predictor**	***B***	**95% CI**	***R*^**2**^**
1	Suicidal ideation	Psychotic experiences	0.092^**^	[0.082,0.101]	0.439
2	Depressive symptoms	Psychotic experiences	0.469^**^	[0.441,0.498]	0.627
3	Suicidal ideation	Depressive symptoms	0.101^**^	[0.067,0.106]	0.523
4		Psychotic experiences	0.044^**^	[0.033,0.055]	

** *p < 0.001*.

The additional analyses exploring anxiety symptoms (AS) and stress (S) separately as single mediators between PE and SI yielded that both AS (b = 0.168, 95% BCa CI [0.128, 0.212]) and S (b = 0.15, 95% BCa CI [0.108, 0.192]) had a significant indirect effect (Supplementary Tables 2, 3). However, the multiple mediation analysis revealed that DS was the only mediator that remained similar and stable in both analyses (b = 0.195, 95% BCa CI [0.137, 0.254]). S did not longer mediate the association (b = −0.009, 95% BCa CI [−0.061, 0.046]); AS did show a mediating effect, but this was a very small indirect effect (b = 0.049, 95% BCa CI [0.022, 0.254]) (Supplementary Table 4).

### Network Analysis

**Figure 2:**
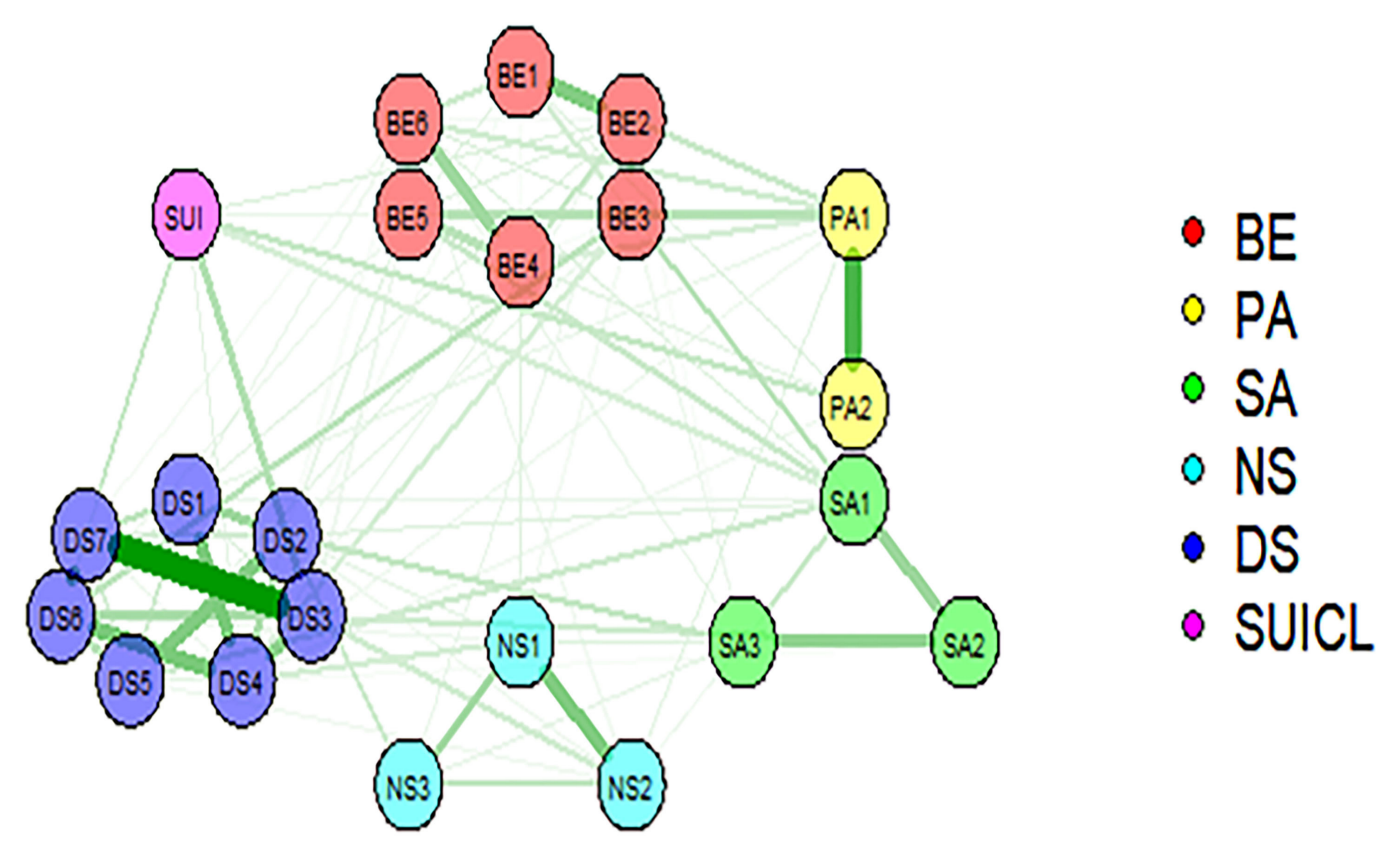
Representation of network model of psychotic experiences, suicidal ideation, and depressive symptoms (BE, bizarre experiences; PA, perceptual anomalies; SA, social anxiety; NS, negative symptoms); DASS, depressive symptoms, SUICL, suicidal ideation (life time). Two main components are depicted: the symptoms or nodes (circles), and the edges (lines linking the symptoms). The edges represent the relationships between symptoms (green lines correspond to positive associations). The thickness of the edges represents the magnitude of the associate ion between nodes.

Figure 2 presents results of the Ising analysis. As expected due to the clustering of items within each instrument, there are more connections within each domain than between domains. The results show different connectivity patterns within both domains of DS and PE, and differential connections among the nodes belonging to these clusters and SI. The strongest within-domain interconnectedness was observed for DS. Within DS, the strongest link was found between DASS3 (“I felt I had nothing to live for”) and DASS7 (“I felt life had no meaning”). Within PE, the strongest link was observed between auditory perceptual anomalies (PA1: “hearing voices when you are alone?”) and visual perceptual anomalies (PA2: “seeing objects, people or animals than others cannot?”).

Regarding the connectedness between different clusters of PE, the strongest links were between PA1 (“hearing voices when alone?”) and bizarre experiences (BE5: “thoughts in your head are not your own?”), followed by BE3 (“people look at you oddly because of your appearance?”) and social anxiety (SA1“I cannot get close to people”), and between BE3 and DASS6 (“I felt I wasn't worth much as a person”). Furthermore, we observed links between DASS3 and NS2 (“I feel mentally insufficient and easily fatigued while thinking or reading”), and between BE3 and DASS4 (“I felt down-hearted and blue”) Supplementary Figure 1 shows differences between edges that were non-zero in the estimated network.

Suicidal ideation was associated with four depressive symptoms (DASS3, DASS7, DASS6, and DASS4), and with several PE: two perceptual anomalies (PA2, PA1), one social anxiety symptom (SA1, “I feel I cannot get close to people”), and two bizarre experiences (BE2 and BE3). The strength values of these associations are shown in Table 7.

**Table 7:** Strenght values of node-specific connections.

**Connections**	**Strength value**
SI-BE2	0.10
SI-BE3	0.16
Mean	0.13
SI-PA1	0.20
SI-PA2	0.51
Mean	0.35
SI-SA1	0.36
SI-DASS3	0.71
SI-DASS4	0.25
SI-DASS6	0.30
SI-DASS7	0.50
Mean	0.44

#### Strength Centrality Index

**Figure 3:**
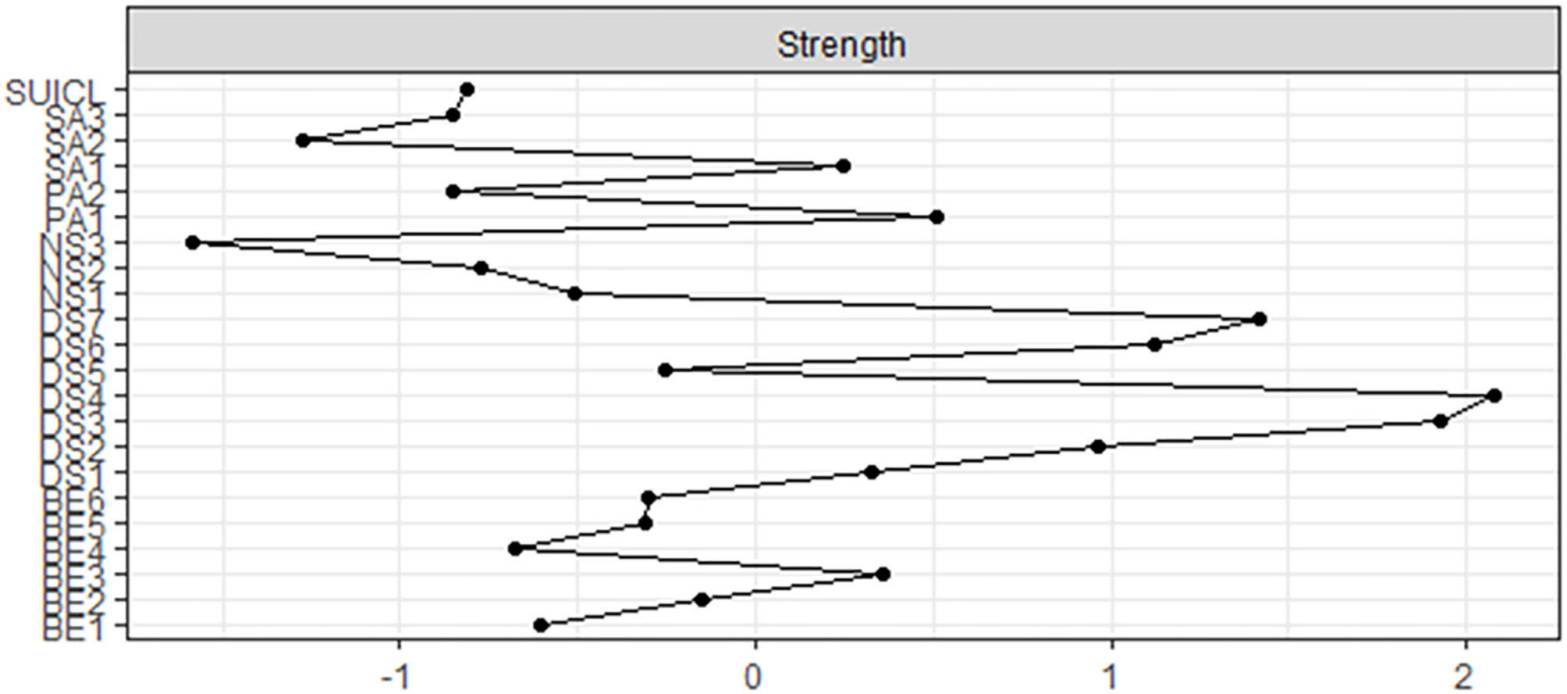
Strength centrality values of nodes per subscale.

As can be seen in Table 8 and Figure 3, DS displayed higher strength values. The strongest nodes in the network were DASS4 (“I felt down-hearted and blue”), DASS3 (“I felt I had nothing to live for”), DASS7 (“I felt life had no meaning”), DASS6 (“I felt I wasn't worth much as a person”), and DASS2 (“It was difficult for me to motivate myself to do things”).

**Table 8:** Mean strength of the 6 subscales.

**Domain**	**Mean**	**Variance**	**Median**
Depressive symptoms	5.71	1.13	5.76
Perceptual anomalies	4.11	1.51	4.11
Bizarre experiences	3.97	0.22	3.94
Social anxiety	3.53	0.99	3.25
Suicidal ideation, lifetime	3.29	NA	3.29
Negative symptoms	3.11	0.55	3.35

#### Network Stability

Supplementary Figure 2A depicts the stability of the centrality indices. The correlation stability coefficient for the strength was 0.60 which is good [63], making our results well interpretable. Supplementary Figure 2B shows the network accuracy, again suggesting our results are acceptable and interpretable. The edge-weight accuracy indicated that most edges are not meaningfully different from each other because their confidence intervals overlap.

## Discussion

We analyzed the associations between psychotic experiences (PE), depressive symptoms (DS) and suicidal ideation (SI) in a large general population sample of adolescents using two complementary approaches: first, we investigated the associations between the three domains in general and second, at item level. The domains of PE, DS, and SI were associated with each other, in accordance with previous literature (61). Confirming previous findings of Sullivan et al. (62), our results suggest that depressive symptoms play an important mediating role in the relationship between SI and PE. Our findings of higher centrality indices for some DS in combination with the results yielded by the mediation analysis, show that the predictive role of PE for suicidal risk in adolescents is not only due to PE themselves. Specifically, our results suggest that these mediating effects may be due to associations between specific aspects of SI, depressive symptoms and psychotic experiences. We found that DS mediated the association through the symptoms of avolition, pessimism, sadness, and feelings of worthlessness that had higher strength values.

These results are consistent with earlier evidence suggesting that some DS are more relevant than others in terms of their impact on impairments and relationships with risk factors (63), and that DS are differentially associated to specific impairments in children and adolescents (64).

In line with by Barragan et al. (65), we found specific associations between PE and DS. We observed links between paranoid ideation and anhedonia, sadness and self-depreciating feelings, and also specific connections between thought alienation/broadcasting symptoms and pessimism, avolition, anhedonia, and self-depreciating feelings. These results, and the specific link observed between auditory hallucinations and pessimism mirror those of another recent study showing that, in patients with mood disorders, only some types of PE (i.e., “hints and double meanings,” “odd looks,” and “being persecuted”) are linked to some depressive symptoms (i.e., “self-criticalness” and “worthlessness”) (30). However, because our cross-sectional design and different populations, we cannot test the directionality of the effects, which remains a critical research question (66).

Previous network analyses examining DS and SI have shown loss of interest, sadness, fatigue and feeling guilty as the most central symptoms for suicidality presentation in adult patients (43). These findings partially align with ours; however, given the differences observed between adult and adolescent depression (67), further specific comparisons using similar samples are needed. Mullarkey et al. (46) is the only study analyzing DS in non-clinical adolescent samples using network analyses. They found that self-hatred, loneliness, sadness, and pessimism were the most central symptoms. Our results are, again, partially in line with these findings, as the four DS that were the most central nodes in our network (“downhearted and blue”; “nothing to look forward to”; “life was meaningless”; and “not being worth much as a person”) are similar to them. Although the age ranges of both samples are similar, direct comparisons must be interpreted with caution, considering we analyzed a narrower range of DS. Our results are also partially consistent with other recent network analysis conducted with adolescent patients reporting that in patients with both major depressive disorder (MDD), social phobia and (SP) and MDD+SP, poor self-esteem and suicidal ideation were the most central, in addition to feelings of worthlessness and anhedonia in the MDD+SP subgroup (68), and with another study reporting that fatigue and low mood as the most central nodes (69). Because the different sample characteristics (non-help seeking adolescents vs. clinical samples), direct comparisons must be cautionary done.

Regarding the associations between PE and SI, the PE domain of social anxiety was connected to SI through the node addressing difficulties to be close to people (SA1), which was clearly linked to the other two social anxiety symptoms, and to bizarre experiences reflecting both paranoid ideation and delusional experiences of thought alienation and thought broadcasting. SA1 was also connected to DS of anhedonia, and slightly associated with avolition. This fits with results from Jaya et al. (70), who found that DS mediated relationships between loneliness and positive symptoms in adults. In addition, they found that loneliness was uniquely associated with paranoid symptoms that were similar to the ones we found to be linked to sadness and feelings of life being meaningless.

Our findings also support previous research showing positive associations between odd beliefs and depressive symptoms relating to negative feelings and sadness in healthy young-adults and adults (29). Although we did not address either social functioning or social isolation, the connections we observed between social anxiety symptoms, bizarre experiences, depressive and negative symptoms are consistent with evidence demonstrating high co-morbidity and strong links between social isolation and loneliness and social anxiety in psychosis (71), between positive PE and perceived social isolation (72, 73) and between PE (delusional mood) and loneliness in a general population sample (20). As argued by Jang et al. (74), PE, and in particular beliefs that other people intend to harm and delusional experiences of thought control/broadcasting, could lead to an inaccurate interpretation of the world, especially in social situations. This potential reality distortion might make individuals less likely to seek out social company, which in turn could increase feelings of worthlessness, meaninglessness and existential void, which were the symptoms that were central in our network. Overall, our findings highlight the need of further research examining the prominent role of loneliness and its relationships with social anxiety and PE, which are regarded as potential predictors of suicidal behavior (75) in school-based sample of adolescents (76), early adolescent patients (77), and young adults with social anxiety disorder and high levels of PE (78).

There are some limitations that should be kept in mind regarding the interpretation of our results. First, regression-based mediation analyses do not explicitly control for measurement error, possibly hindering the adequate estimation of coefficients (79). Second, the meaning of centrality indices in psychology research is not undisputed (59). For instance, central nodes in cross-sectional data were not necessarily the most important symptoms, as found in subjects with social anxiety disorder (80). Finally, cross-sectional networks do not provide information on how symptoms trigger each other over time (81) and thus, causal relationships among symptoms cannot be inferred (96). Furthermore, cross-sectional data only allow examining mechanisms at group level but not at individual level (82). These issues are crucial to investigate further, e.g., through the “suicidal drive hypothesis for psychosis” framework, which states that psychosis could be consequential to suicidal behavior instead of causing higher suicidal risk (83). Finally, we addressed a narrow range of depressive symptoms and did not include some symptoms highlighted as relevant in adolescents when compared to adults (i.e., appetite and weight change and insomnia) (67). Strengths of our study include the large sample size, the use of two complementary analytic methods, and the high stability and moderate accuracy of our analyses. Working from the notion of psychosis existing as an extended phenotype (84), the exploration of subclinical expressions of psychosis offers opportunities to investigate mechanisms that, may explain a vulnerability for the development of psychosis along this continuum” (85). However, it should be kept in mind that this was a general (non-clinical) population sample and generalizability may be limited to more clinical expressions of psychosis.

In our sample, a relevant proportion of adolescents have experienced PE at least sometimes during their lifetime. Comparisons with previous research show different results. For instance, the endorsement rates of BE was lower when compared with Armando et al. (78) and Wüsten et al. (86), but similar when compared with Isaksson et al. (9). Additionally, the rates of PA were similar when compared to these studies and also with Issackson et al. (9), but slightly higher than Narita et al. (20). Because the different measures, sample sizes and age-ranges, clear conclusions on cultural differences cannot be drawn and further research is needed.

Some clinical implications can be drawn from our results. Aligning with other evidence revealing that relationships between PE and SI reflect a higher underlying risk of suicidal behavior as a function of psychiatric symptoms or mental distress (21, 22), our results suggest that youth clinical services should screen for a broad range of symptoms and suicide correlates when assessing suicidal risk. Moreover, our results corroborate the notion that PE play an important role already in early stages of mental health problems in young people (66), causing severe distress (86), higher use of mental health services (87) and reduced functioning, even when transient (88). Despite this evidence, strategies to both address and treat PE in mental health services are not commonly employed (89). Because these strategies may prevent PEs from becoming persistent PE (90) which increases the risk for later mental health problems (91), timely detection of PE should be routinely included in mental health services (10).

## Data Availability Statement

The raw data supporting the conclusions of this article will be made available by the authors, without undue reservation.

## Ethics Statement

The studies involving human participants were reviewed and approved by Bioethics Committee of University of Talca. Written informed consent to participate in this study was provided by the participants' legal guardian/next of kin.

## Author Contributions

DN designed the study and directed its implementation, did the literature search, and wrote the manuscript. PM did the literature search and performed preliminary network analyses. SC performed the mediation analyses, edited, and reviewed the manuscript. JW reviewed the manuscript and revised it critically for intellectual content. All authors contributed to the article and approved the submitted version.

## Conflict of Interest

The authors declare that the research was conducted in the absence of any commercial or financial relationships that could be construed as a potential conflict of interest.
