# Supplementary material for: Evidence for Specific Associations Between Depressive Symptoms, Psychotic Experiences, and Suicidal Ideation in Chilean Adolescents From the General Population
Source: Front Psychiatry. 2021 Jan 28;11:552343. doi: 10.3389/fpsyt.2020.552343 (PMC7876080; doi:10.3389/fpsyt.2020.552343)

Supplementary material

**Supplementary Figure 1.** Bootstrapped difference tests (<0.05) between edge-weights that were non-zero in the estimated network. Gray boxes indicate nodes or edges that do not differ significantly from one-another and black boxes represent nodes or edges that do differ significantly from one-another.

**Supplementary Figure** **2**. A) Average correlations between centrality indices of networks sampled with persons dropped and the original sample. Lines indicate the means and areas indicate the range from the 2:5th quantile to the 97:5th quantile. B) Bootstrapped confidence intervals of estimated edge-weights for the estimated network of PE, SI and DS. The red line indicates the edge weight values and the gray area the 95% CIs.

Supplementary Table 1: **Correlation among variables**

|  | SI | PE | BE | PA | SA | NS |
| --- | --- | --- | --- | --- | --- | --- |
| SI |  |  |  |  |  |  |
| PE | .436** |  |  |  |  |  |
| BE | .412** | .825** |  |  |  |  |
| PA | .314** | .516** | .446** |  |  |  |
| SA | .221** | .668** | .361** | .159** |  |  |
| NS | .243** | .672** | .370** | .204** | .319** |  |
| DS | .493** | .634** | .512** | .337** | .443** | .443** |

PE= Psychotic experiences (Total); BE= Bizarre experiences; PA= Perceptual anomalies; SA= Social anxiety; NS= Negative symptoms; DS= Depressive symptoms.

*p<.005; **p<.001

Supplementary Table 2. **Mediation analysis of psychotic experiences, anxiety and suicidal ideation.**

| Mediation steps | Outcome | Predictor | *B* | 95% CI | *R*^2^ |
| --- | --- | --- | --- | --- | --- |
| 1 | SI | PE | .091** | [.082, .1.01] | 0.193 |
| 2 | AS | PE | .396** | [.372, .421] | 0.387 |
| 3 | SI | AS | .088** | [.060, .106] | 0.238 |
| 4 |  | PE | .056** | [.045, .068] |  |

Supplementary Table 3. **Mediation analysis of psychotic experiences, stress and suicidal ideation.**

| Mediation steps | Outcome | Predictor | *B* | 95% CI | *R*^2^ |
| --- | --- | --- | --- | --- | --- |
| 1 | SI | PE | .092** | [.082, .1.01] | 0.193 |
| 2 | S | PE | .432** | [.405, .459] | 0.377 |
| 3 | SI | S | .072** | [.056, .088] | 0.230 |
| 4 |  | PE | .060** | [.049, .072] |  |

Supplementary Table 4. **Multiple mediation analysis of anxiety, stress and depressive symptoms on the link of psychotic experiences and suicidal ideation**

| Mediation steps | Outcome | Predictor | *B* | 95% CI | *R*^2^ |
| --- | --- | --- | --- | --- | --- |
| 1 | SI | PE | .090** | [.080, .098] | 0.191 |
| 2 | DS | PE | .469** | [.441, .479] | 0.393 |
| 3 | AS | PE | .396** | [.372, .421] | 0.387 |
| 4 | S | PE | .432** | [.405, .459] | 0.377 |
| 5 | SI | DS | .086** | [.067, .106] | 0.277 |
| 6 | SI | AS | .036* | [.013, .060] | 0.277 |
| 7 | SI | S | -0.004 | [-.026, .017] | 0.277 |
| 8 |  | PE | .038** | [.026, .050] |  |

Supplementary Figure 1.

Supplementary Figure 2.


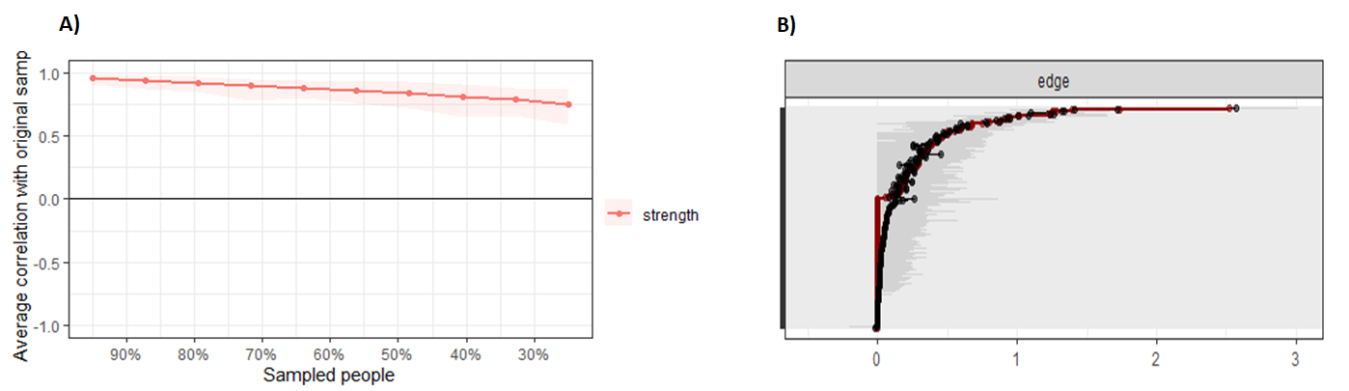

Supplement: Supplementary file 1 [file Table_1.docx]
